# Differences in park characteristic preferences for visitation and physical activity among adolescents: A latent class analysis

**DOI:** 10.1371/journal.pone.0212920

**Published:** 2019-03-18

**Authors:** Lieze Mertens, Jelle Van Cauwenberg, Jenny Veitch, Benedicte Deforche, Delfien Van Dyck

**Affiliations:** 1 Department of Movement and Sport Sciences, Faculty of Medicine and Health Sciences, Ghent University, Ghent, Belgium; 2 Research Foundation Flanders (FWO), Brussels, Belgium; 3 Department of Public Health, Faculty of Medicine and Health Sciences, Ghent University, Ghent, Belgium; 4 Institute for Physical Activity and Nutrition (IPAN), Deakin University, Geelong, Australia; 5 Physical Activity, Nutrition and Health Research Unit, Faculty of Physical Education and Physical Therapy, Vrije Universiteit Brussel, Brussels, Belgium; Guangzhou University, CHINA

## Abstract

In order to optimize environmental interventions, the current study aimed to investigate whether there are subgroups with different preferences regarding park characteristics for park visitation and park-based PA among adolescents (12–16 years). Furthermore, we examined whether the identified subgroups differed in socio-demographics, PA behavior, and park use characteristics (e.g. accompaniment to park, usual activities during park visitation, usual transportation to parks). Adolescents (12–16 years) were recruited via randomly selected secondary schools, located in Flanders (Belgium). Class visits were conducted between September and November 2016 and adolescents were asked to complete an online questionnaire. Latent class analyses using Sawtooth Software were used to identify possible subgroups. A final sample of 972 adolescents (mean age 13.3 ± 1.3 years) remained for analyses. Three subgroups of adolescents with similar preferences for park characteristics could be distinguished for both park visitation and park-based PA. Overall, current results indicate that park upkeep was the most important park characteristic for park visitation as well as park-based PA among at risk subgroups (i.e. adolescents with lower overall PA levels, girls, older adolescents,…) followed by the presence of a playground or outdoor fitness equipment. Among the more active adolescents, especially boys visiting the parks together with friends, the presence of a sport field (soccer and basketball) seems to be the best strategy to increase park visitation as well as park-based PA. Current results provide a starting point to advise policy makers and urban planners when designing or renovating parks that investing in good upkeep and maintenance of parks, and the provision of a playground or outdoor fitness equipment might be the best strategy to increase both park visitation and park-based PA among at risk adolescent subgroups.

## Introduction

Globally, almost half of adolescents are insufficiently physically active and do not achieve the public health guideline of 60 minutes/day moderate- to vigorous-intensity physical activity (PA) [1–3]. Moreover, the prevalence of overweight and obesity among adolescents is continuously rising [4,5]. Sufficient PA can improve adolescents’ physical and mental health [6–8], and can prevent the development of obesity and non-communicable diseases such as cardiovascular diseases and type 2 diabetes [9]. As higher levels of PA during adolescence can lead to higher PA levels during adulthood [10,11], it is important to promote adolescents’ PA at the population level.

Socio-ecological models emphasize the importance of physical environmental factors (e.g. accessibility of recreation facilities, quality of park facilities) in addition to individual or social factors (e.g. socio-demographics, social support) to explain PA levels among youth [12]. These models incorporate the four domains of active living; active recreation, active transport, household activities and occupational activities [13]. Within the domain of active recreation, existing literature has identified the importance of parks for adolescents to accumulate PA [14–16]. However, previous research has also indicated that low numbers of adolescents are visiting parks [17,18]. It is therefore important to understand which physical and social park characteristics [19] may attract adolescents to visit and be physically active in parks.

Previous cross-sectional qualitative and quantitative research among adolescents in Belgium [20] has revealed several park characteristics related to park visitation or park-based PA. For example, the presence of greenery [21,22], open spaces [19], sport and play facilities [19,23,24], and the presence of other adolescents and friends [19,25] were positively associated with park visitation or park-based PA. However, stronger designs with improved causal inference are needed. Natural experiments (i.e. park renovations) in the US [26] and in Australia [27,28] have already shown that improving specific park characteristics such as fencing, the installation of a new walking path and improvements in landscaping can lead to increased park visitation and park-based PA. Nonetheless, natural experiments are still scarce because they are usually long-term and expensive projects. Furthermore, it is not defensible to change real environments without being sure that these changes are effective [29].

Therefore, we developed a cost-effective and efficient methodology using manipulated photographs [30,31] to simulate natural experiments and to investigate associations between park-based physical and social environmental factors and park’s appeal for visitation and PA. A recent large-scale experimental study using manipulated photographs among adolescents identified the most important park characteristics influencing the appeal for park visitation and park-based PA [32]. The main finding was that better park maintenance (i.e. good park upkeep) was the most important characteristic for both park visitation and park-based PA, followed by the presence of playground/outdoor fitness equipment and sport fields. However, it remains unclear whether these park characteristics are more or less important for specific subgroups of adolescents (e.g. boys versus girls, younger versus older adolescents, low versus high social status, frequent park visitors versus irregular visitors, often accompanied by friends versus not often accompanied by friends) [33]. To maximize park visitation and park-based PA, it may be essential to target infrastructure and policies that are most likely to reach at-risk subgroups (i.e. those that are currently not visiting or being physically active in parks). It is known that girls [34–36], older adolescents [33,37], and adolescents with lower parental educational levels [34,36,38] or family income [38] have lower overall PA levels. Moreover, parks may be an opportune setting to reach minority groups (such as low SES and non-western-European adolescents) that are hard to reach and are at risk for physical inactivity [39]. Moreover, PA in parks may increase social cohesion and integration of minority youth into society and enhance mental health and social interactions [40]. Previous research has suggested that the company of friends and the presence of active peers are associated with higher levels of PA among adolescents [41,42].

Consequently, in order to optimize environmental interventions aiming to encourage park visitation and park-based PA, it is important to investigate which park characteristics are specifically more or less important for particular subgroups based on socio-demographic factors, PA behavior, and park use characteristics (e.g. accompaniment to park, usual activities during park visitation, usual transportation to parks). Lastly, it is important to understand which park characteristics are important for both park visitation and park-based PA as these different behaviors may be influenced by different park characteristics [32]. For example, a park with benches was preferred for park visitation while a park without benches was preferred for PA or sedentary peers were preferred over no peers for park visitation, whereas for park-based PA no peers were preferred over sedentary peers [32].

Therefore, the current study aimed to investigate whether there are subgroups with different preferences regarding park characteristics for park visitation and park-based PA among adolescents (12–16 years) using latent class analysis. Furthermore, we examined whether the identified subgroups differed in socio-demographics, PA behavior, and park use characteristics (e.g. accompaniment to park, usual activities during park visitation, usual transportation to parks). Based on existing, mainly qualitative literature, we hypothesized that for adolescent girls, naturalness [43]; constructed walking paths [43], feeling safe (i.e. fear from strangers) [44,45] and providing facilities for individual, non-competitive ‘fun’ activities might be important for their park visitation or park-based PA [46–48], while for adolescent boys, the presence of a sport field might be most important [49].

## Methods

### Study design and sampling

Adolescents (12–16 years) were recruited via randomly selected secondary schools, located in the province Flemish-Brabant (Flanders, Belgium). The sampling of schools and recruitment of participants have been described in detail elsewhere [32]. Briefly, from the 103 contacted schools, more than half (61.2%) did not respond and 30 schools (29.1%) declined participation with reasons being; too many requests for research (n = 11), not interested (n = 10) and no time (n = 9). Ten schools agreed to participate (response rate = 9.7%) and were asked to select at least two classes from grade one to grade four (ages 12–16). Adolescents indicated their consent by signing an informed consent (i.e. active written consent), while parents were given the opportunity to refuse their child’s participation by returning a form to the school. Without refusal, consent was assumed (i.e. passive consent). This approach was selected as the questionnaire was anonymous and involved a non-sensitive topic [50,51], this approach was selected. School visits were conducted between September and November 2016 and adolescents were asked to complete an online questionnaire during class time. The study protocol and the research protocol for minors was approved by the Ethics Committee of the University Hospital of Ghent University (2016/0284), referring to the privacy act of December 8th, 2012 on the protection of privacy in relation to the processing of personal data [52].

### The online questionnaire

The online questionnaire was developed with Sawtooth Software (Lighthouse studio 9.2.0.) and consisted of two parts: a questionnaire about participant characteristics and two sets of ten randomly assigned choice tasks using manipulated photographs developed with Adobe Photoshop software (see S1 Additional file (Questionnaire Dutch version) and S2 Additional file (Questionnaire English version). In the first part, questions gathered information about participants’ socio-demographics, PA levels, and park use. Socio-demographic variables included age, sex, school, grade, height, weight, residence (urban, suburban, rural), health (are you healthy enough to be physically active), highest level of parental education, nationality, ethnicity (place of birth of the adolescent, mother and father), and socio-economic status (SES). Ethnicity was based on the definition of the Flemish government [53] which assigns a foreign origin to someone having at least one parent born outside of the EU15. SES was assessed by six items of the Family Affluence Scale (FAS) and categorized into low (FAS score 0–6), medium (FAS score 7–9), and high SES (FAS score 10–13) by calculating the total score of FAS minus 6 [54]. PA levels were assessed using the validated Flemish Physical activity Questionnaire (FPAQ) [55], which has been previously used to assess the PA levels among adolescents [56–58]. Lastly, questions about park use were derived from questions previously used by Veitch et al. [59]: frequency of visitation in the last three months, average duration of visitation in the last three months, usual accompaniment to parks, activities usually performed in the park, and transportation to the park. S1 Table (Relative importances of each park characteristic for park visitation, socio-demographics, PA behavior and park use characteristics for the three subgroups identified by latent class analysis (complete table)). provides a detailed overview of all possible response categories.

**Fig 1:**
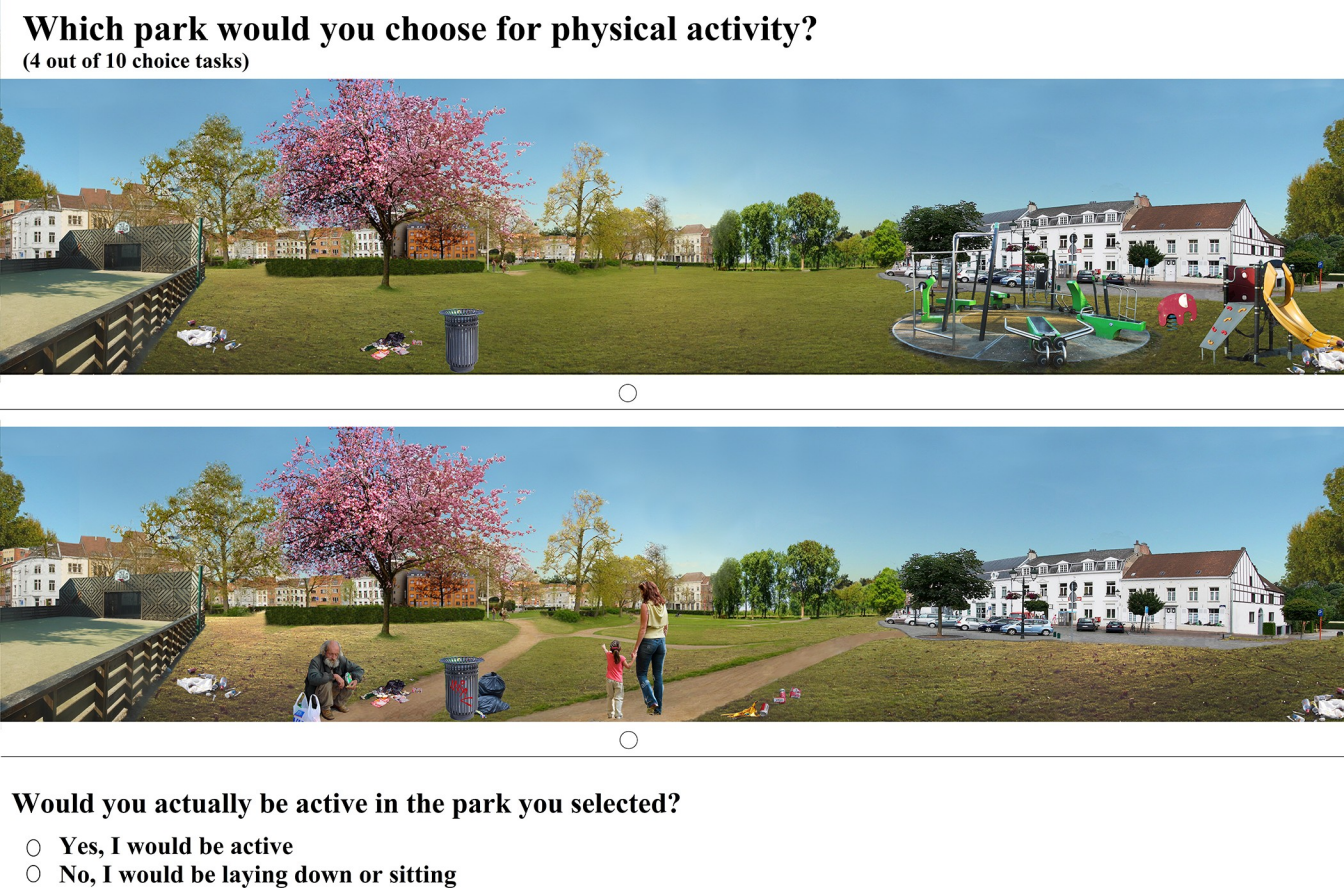
Example of choice task in the second set of choice tasks.

In the second part of the web-based questionnaire, two sets of ten choice-based conjoint (CBC) tasks were presented to participants using manipulated photographs to illustrate two different parks. For both sets, the same manipulated photographs were used but the research purpose was different. For the first set, participants had to choose which of the two depicted parks they would prefer to visit, while for the second set, they had to select which park they would choose for PA. PA was clarified as: *“all activities except sitting and laying down*, *such as playing active games*, *walking the dog or sports such as soccer”*., After selecting the park they would choose for PA, participants were asked if they would actually be active in the park they selected?” (Fig 1).

A detailed description of the photograph development, manipulation process and the specific levels of each characteristic can be found elsewhere [32]. Briefly, ten park characteristics were carefully selected based on previous qualitative research [19] and existing literature [23,25,60–63]. Since the space on the photographs was limited, only a small number of park characteristics could be included in each photograph. First, park characteristics shown to be associated with park visitation or park-based PA among adolescents in quantitative research [20], were prioritized for inclusion. Second, characteristics identified in our previous qualitative study [19] were also selected. Characteristics that are difficult to manipulate in photographs (e.g., sufficient lighting in the evening) were not included. The following ten park characteristics, each varying in two to four levels were manipulated in this study: naturalness (i.e. plants and trees) (2 levels), walking paths (3 levels), upkeep (3 levels), outdoor fitness equipment/playground (4 levels), sport field (2 levels), benches (2 levels), drinking fountain (2 levels), peers (3 levels), mother with a child (2 levels) and homeless person (2 levels). For example, upkeep of the park was depicted in three levels: poor maintenance (graffiti, trash, poorly maintained grass field), moderate maintenance (no graffiti, some trash, moderately maintained grass field) and good maintenance (no graffiti, no trash, good maintained grass field). Each manipulated photograph differed in at least one park characteristic, yielding a total of 6912 manipulated photographs. The selection of these park characteristics was based on previous qualitative research [19] and existing literature [23,25,60–63].

### Analyses

SPSS Statistics 24 was used to calculate the descriptive characteristics of the sample, and Sawtooth Software (Lighthouse Studio 9.2.0) was used to perform the latent class analyses [64]. Conjoint analyses do not accommodate ‘typical’ moderation analysis, but latent class analysis can be used to distinguish various subgroups according to their park characteristic preferences based on the choice-based conjoint tasks [64,65]. The cluster criterion choice used by latent class analysis is less arbitrary than the standard cluster analysis and consequently shows a higher construct and predictive validity [66–68]. Latent class analysis assigns each participant to a subgroup based on the highest probability of belonging to a class [69]. The final number of subgroups (n = 3) was selected based on the model fit and the number of participants in each subgroup [64]. In S3 Additional file (A detailed overview of the different models for two, three, four and five subgroups is given for park visitation) and S4 Additional file (A detailed overview of the different models for two, three, four and five subgroups is given for park-based PA), a detailed overview of the different models for two, three, four and five subgroups is given. For park visitation, the model of three subgroups had a distribution of respectively 666 (68.6%), 147 (15.1%) and 158 (16.3%) participants in each subgroup. For park-based PA, there was a distribution of respectively 341 (35.2%), 153 (15.8%) and 476 (49.1%) participants in each subgroup.

For each subgroup separately, Hierarchical Bayes (HB) estimation using dummy coding was executed to calculate the average relative importances and 95% confidence intervals of the different park characteristics [70]. These average relative importances represent the influence of each park characteristic on the preference for park visitation/park-based PA. Relative importances with non-overlapping 95% confidence intervals differ significantly from each other [65]. Furthermore, chi-square analyses (categorical variables) and MANOVAs (continuous variables) with Scheffe post-hoc analyses were performed in SPSS Statistics 24 to examine the significant differences in socio-demographics, PA behavior, and park use characteristics between the various subgroups. For all analyses, statistical significance was set at p < 0.05.

## Results

### Descriptive statistics

After data cleaning, a final sample of 972 adolescents remained for analyses (see S1 Dataset). A detailed description of the total sample can be found elsewhere [32]. The mean age was 13.3 ± 1.3 years, 54.0% were girls, 64.9% were in the first or second grade, 77.6% had at least one parent with higher education and 45.7% of participants complied with the guidelines of 60 minutes moderate-to-vigorous intensity PA (MVPA) daily. All following results are shown separately for park visitation and for park-based PA.

### Subgroup analysis–park visitation

Regarding park visitation, latent class analysis revealed three subgroups with homogenous preferences for park characteristics. Table 1 presents the relative importance (i.e. the relative magnitude of effect) of each park characteristic on the choice to visit a park, within each subgroup and also the significant differences in socio-demographics, PA behavior and park use characteristics. A complete table (i.e. including also the non-significant differences) can be found in S1 Table (Relative importances of each park characteristic for park visitation, socio-demographics, PA behavior and park use characteristics for the three subgroups identified by latent class analysis (complete table)).

**Table 1:** Relative importances of each park characteristic for park visitation, socio-demographics, PA behavior and park use characteristics for the three subgroups identified by latent class analysis.

	Subgroup 1	Subgroup 2	Subgroup 3	*p-value*
**Subgroup Sizes (n)**	n = 666	n = 147	n = 158	
	68.6%	15.1%	16.3%	
**Relative Importances (M (95% CI))**				
Upkeep	48.1 (47.5,48.6)	25.7 (24.1,27.2)	26.2 (24.6,27.8)	
Playground/outdoor fitness	15.3 (14.9,15.8)	14.6 (13.5,15.6)	21.1 (19.7,22,6)	
Sport field	8.0 (7.6,8.4)	30.2 (28.8,31.6)	9.3 (8.4,10.3)	
Activity peers	5.0 (4.7,5.2)	6.5 (5.8,7.2)	11.3 (10.2,12.3)	
Homeless person	5.6 (5.4,5.9)	4.3 (3.7,4.8)	8.2 (7.2,9.3)	
Walking paths	5.4 (5.2,5.6)	6.1 (5.7,6.6)	6.9 (6.3,7.5)	
Naturalness	4.5 (4.3,4.7)	4.9 (4.3,5.4)	6.3 (5.6,7.1)	
Mother with a child	3.4 (3.3,3.6)	2.6 (2.3,3.0)	3.4 (3.0,3.8)	
Benches	2.4 (2.2,2.5)	2.8 (2.5,3.2)	4.1 (3.6,4.6)	
Drinking fountain	2.2 (2.1,2.4)	2.4 (2.1,2.7)	3.1 (2.7,3.5)	
**Socio-demographic characteristics**				
Age (years, M±SD)	13.2 ± 1.2 ^b^	13.5 ± 1.4 ^a^	13.4 ± 1.3	0.015*
Gender (% women)	61.6 ^b^	20.4 ^a^^,^^c^	53.8 ^b^	<0.001*
Other ethnicity (%)	31.1 ^b^^,^^c^	44.2 ^a^	46.8 ^a^	<0.001*
Education (% at least one parent high educated)	79.1 ^c^	81.4 ^c^	66.7 ^a^^,^^b^	0.011*
Living area (%)				0.007*
- Rural	11.1	7.5	10.8	
- Suburban	60.5	49.7	52.5	
- Urban	28.4	42.9	36.7	
Meets PA guidelines (%)	43.1	58.5	44.9	0.003*
Member of sport club (%)	67.7	79.6	57.6	<0.001*
How many friends do you have?	6.8 ± 9.1	11.6 ± 15.3	8.9 ± 12.3	<0.001*
**PA behavior (min/week)**				
Moderate-to-vigorous intensity PA (M±SD)	438.6 ± 367.2 ^b^	569.3 ± 390.0 ^a^^,^^c^	443.7 ± 382.0 ^b^	0.001*
**Park use characteristics**				
Frequency of visitation (M±SD)	3.5 ± 1.7	3.8 ± 1.9	3.8 ±1.8	0.032*
Park duration (min/3 months) (M±SD)	75.3 ± 57.1 ^b^	96.3 ± 68.3 ^a^	85.2 ± 64.4	0.001*
Walking distance to closest park (min) (M±SD)	14.0 ± 12.3	12.2 ± 11.8	11.7 ± 11.0	0.058
**Accompaniment to parks**				
Friends (%)	60.5	76.7	61.0	0.002*
Parents/grandparents/aunt/uncle (%)	38.6	14.7	29.1	<0.001*
Dog (%)	16.0	7.0	7.8	0.003*
**Usual activities during park visitation**				
Walking (%)	67.8	34.1	53.2	<0.001*
Ball sports (%)	37.7	72.9	39.7	<0.001*
Sitting/lying down (%)	39.8	27.9	34.0	0.029*

^a^ significant difference with subgroup 1

^b^ significant difference with subgroup 2

^c^ significant difference with subgroup 3

* p < 0.05

The first subgroup, which represented the majority of all respondents (68.6%), distinguished itself from the other subgroups by awarding most importance to park upkeep. They were more likely to be accompanied to the park by (grand)parents/aunt/uncle or by a dog, walk or sit or lay down when in the park and visit for a shorter duration compared to both other subgroups. This subgroup, consisted mainly of adolescent girls from a Belgium ethnicity with a highly educated parent, living in suburban areas.

The second subgroup (representing 15.1% of participants) paid relatively more importance to the presence of a sport field compared to both other subgroups, followed by upkeep and the presence of a playground or outdoor fitness equipment. This subgroup, consisted mainly of boys, they reported having the most friends, were most likely to be accompanied to the park by friends, engaged more in ball sports while visiting the park, visited for a longer duration, and were more likely to be a member of a sport club compared to both other subgroups. They also reported significantly higher levels of moderate-to vigorous PA and were the most active subgroup (i.e. almost 60% met the PA guidelines) compared to both other subgroups.

Besides the importance of upkeep, subgroup 3 (16.3%) attached relatively more importance to the presence of a playground or outdoor fitness equipment, and the activity of peers as the most important park characteristics to visit a park. This subgroup consisted of more adolescents from another ethnicity, had less highly educated parents, and showed the lowest percentage of sport club members in comparison to both other subgroups.

No significant differences between the three subgroups were found for place of birth (i.e. born in Belgium), SES, weight-for-age related categories, light PA, walking distance to closest park, and usual transportation to parks in the last three months (see S1 Table).

### Subgroup analysis–Park-based PA

Regarding park-based PA, latent class analysis also revealed three subgroups with homogenous preferences for the park characteristics. Table 2 presents the relative importance (i.e. the relative magnitude of effect) of each park characteristic on the choice to use the park to be physically active, within each subgroup and also the significant differences in socio-demographics, PA behavior and park use characteristics. A complete table (i.e. including also the non-significant differences) can be found in S2 Table (Relative importances of each park characteristic for park-based PA, socio-demographics, PA behavior and park use characteristics for the three subgroups identified by latent class analysis (complete table)).

**Table 2:** Relative importances of each park characteristic for park-based PA, socio-demographics, PA behavior and park use characteristics for the three subgroups identified by latent class analysis.

	Subgroup 1	Subgroup 2	Subgroup 3	p-value
**Subgroups Sizes (n)**	n = 341	n = 153	n = 476	
	35.2%	15.8%	49.1%	
**Average relative importances %** **(M (95% CI))**				
Upkeep (M (95% CI))	44.0 (42.6,45.4)	32.3 (30.3,34.2)	41.9 (40.8,43.0)	
Playground/outdoor fitness	14.5 (13.9,15.2)	15.3 (14.3,16.3)	17.6 (17.0,18.3)	
Sport field	9.1 (8.2,9.9)	23.1 (21.2,25.0)	9.7 (7.0,10.3)	
Activity peers	6.3 (5.8,6.9)	6.0 (5.3,6.7)	6.2 (5.8,6.7)	
Homeless person	6.5 (6.0,7.0)	5.0 (4.4,5.5)	5.7 (5.3,6.0)	
Walking paths	5.8 (5.5,6.1)	5.8 (5.4,6.3)	5.7 (5.5,5.9)	
Naturalness	4.9 (4.6,5.3)	4.8 (4.3,5.4)	4.8 (4.5,5.1)	
Mother with a child	3.4 (3.2,3.7)	2.8 (2.5,3.1)	3.4 (3.2,3.6)	
Benches	2.9 (2.7,3.2)	2.6 (2.2,2.9)	2.6 (2.4,2.8)	
Drinking fountain	2.4 (2.2,2.6)	2.4 (2.1,2.7)	2.4 (2.3,2.6)	
**Socio-demographic characteristics**				
Age (years, M±SD)	13.6 ± 1.2 ^c^	13.5 ± 1.4 ^c^	13.1 ± 1.2 ^a^^,^^b^	<0.001*
Gender (% women)	65.1 ^b^^,^^c^	19.6 ^a^^,^^c^	57.1 ^a^^,^^b^	<0.001*
Meets PA guidelines (%)	33.4	63.4	48.9	<0.001*
Member of sport club (%)	63.9	78.4	67.2	0.006 *
Categories z-scores (BMI) (%)				0.047 *
- Underweight	4.2	4.1	7.9	
- Normal weight	86.4	91.2	58.7	
- Overweight	9.3	4.7	6.4	
**PA behavior (min/week)**				
Light PA (M±SD)	222.6 ± 197.6 ^c^	261.5 ± 232.8	264.2 ± 223.0 ^a^	0.019 *
Moderate-to-vigorous intensity PA (M±SD)	370.6 ± 348.1 ^b^^,^^c^	585.8 ± 360.6 ^a^^,^^c^	482.1 ± 385.3 ^a^^,^^b^	<0.001*
**Park use characteristics**				
Park use (M±SD)	3.3 ± 1.7 ^b^^,^^c^	3.8 ± 1.8 ^a^	3.7 ±1.7 ^a^	0.005*
Park duration (min/3 months) (M±SD)	71.7 ± 53.6 ^b^	88.7 ± 68.8 ^a^	82.9 ± 61.4	0.010*
**Accompaniment to the park**				
Friends (%)	62.4	73.3	60.2	0.022*
(Step)brother/sister/niece/nephew (%)	34.5	25.2	40.0	0.006*
Parents/grandparents/aunt/uncle (%)	33.8	17.0	38.4	<0.001*
**Usual activities during park visitation**				
Walking (%)	69.0	35.6	62.1	<0.001*
Ball sports (%)	31.4	71.9	42.4	<0.001*
Sitting/lying down (%)	43.6	24.4	36.5	0.001*
Jogging (%)	18.5	12.6	24.4	0.007 *
Active games (%)	10.5	5.9	20.1	<0.001*
Exercising (%)	5.9	10.4	15.9	<0.001*
**Usual transportation to parks**				
Public transportation (%)	22.6	15.6	13.5	0.006 *

^a^ significant difference with subgroup 1

^b^ significant difference with subgroup 2

^c^ significant difference with subgroup 3

* p < 0.05

The first subgroup, representing 35.2% of participating adolescents, awarded most importance to park upkeep, followed by the the presence of a playground or outdoor fitness equipment. Subgroup 1 included the highest proportion of girls, visited parks less frequently and for a shorter duration, were more likely to walk, sit or lay down in the park, and were more likely to use public transport to travel to parks.

The second subgroup, represented 15.8% of participating adolescents, and distinguished itself from the other subgroups by awarding relatively more importance to the presence of a sport field. This subgroup consisted mainly of boys, they visited parks more frequently and for a longer duration, engaged more in ball sports when in the park, were more likely to be accompanied by friends while visiting the park and were the most active subgroup (more than 60% met the PA guidelines) in comparison to both other subgroups.

Subgroup 3 (49.1%) indicated similar park characteristic preferences as subgroup 1, but distinguished itself by being the youngest group of adolescents. They were more likely to be accompanied by family, and engaged more in active games or exercising during park visits in comparison to both other subgroups.

No significant differences between the three subgroups were found for place of birth, ethnicity, education, SES, living area, number of friends, engagement in light PA, and walking distance to closest park (see S2 Table).

## Discussion

In order to optimize environmental interventions aiming to encourage park visitation and park-based PA for all users, the different needs of particular subgroups need to be identified. In this paper, three subgroups of adolescents with similar preferences for park characteristics could be distinguished for both park visitation and park-based PA.

Regarding park visitation, less than the half of subgroup 1 (43.1%) and subgroup 3 (44.9%) met the PA guidelines, with both groups achieving significantly less MVPA in comparison to subgroup 2. Moreover, subgroups 1 and 3 consisted of more girls, fewer sport club members, and adolescents with lower parental educational level (i.e. subgroup 3) compared with subgroup 2, which are known from the literature as at risk populations for low overall PA levels [34–36,38]. Furthermore, subgroup 1 visited parks least frequently and reported the shortest park visitation duration compared to subgroups 2 and 3.

Regarding park-based PA, only one-third of subgroup 1 (33.4%) and less than the half of subgroup 3 (48.9%) met the PA guidelines. Subgroup 1 represented an at risk subgroup because this group consisted of adolescent older girls, who visited parks less frequently and for a shorter duration, were more likely to walk, sit or lay down in the park, and were more likely to use public transportation to travel to the parks [33–37]. Subgroup 3, represented the youngest adolescent subgroup, who showed significantly higher MVPA than subgroup 1, but less than subgroup 2, and are also considered an at risk group.

Overall, the current results indicate that park upkeep was by far the most important park characteristic for park visitation as well as park-based PA for subgroups 1 and 3 which were the at risk subgroups. This was followed by the presence of a playground or outdoor fitness equipment. In the literature, there is consistency about the fact that the absence of rubbish and parks with better maintenance, are related to more park visitation and park-based PA [19,59,62,71]. For example, an observational study in the US found that good levels of maintenance and cleanliness are important factors attracting middle-school children to parks [62]. A qualitative study using walk-along interviews in Belgium indicated that unwanted graffiti was not attractive for visiting a public open space (e.g. parks, playgrounds, squares, streets), and that bad upkeep affected adolescents’ actives use of an public open space [19]. An experimental study in Australia revealed that a steep slide (playground equipment) and absence of rubbish/graffiti were the two most important features for park visitation [71]. This may be explained by the positive influence of park upkeep on the perception of aesthetics and safety [19]. To conclude, investing in good upkeep and maintenance of parks, and in the provision of a playground or outdoor fitness equipment might increase park visitation and park-based PA among adolescents at risk of low PA (mainly girls). These findings do not support our initial hypothesis that naturalness, the presence of walking paths and the absence of a homeless person would be most important for girls. This might be due to the fact that the hypotheses were based primarily on qualitative research.

However, in line with our predetermined hypothesis, we found that among the more active adolescents, especially boys visiting the parks together with friends, the presence of a sport field (soccer and basketball) seems to be the best strategy to increase park visitation as well as park-based PA. However in a previous study of Veitch et al. (2017), the presence of basketball courts was only ranked 7th out of 10 attributes that encourage park visitation among adolescents. With this finding, we could demonstrate that analyses by subgroups provides a different perspective and adds to existing literature, i.e. among boys and those who are active courts are more important than when examined not by subgroup. Broadly speaking, for the other park characteristics (i.e. activity peers, the presence of a homeless person, walking paths, naturalness, mother with a child, benches and drinking fountain) no consistent pattern of differences in importance between the subgroups could be distinguished.

Current results contribute existing knowledge about the preferences for park visitation and park-based PA among adolescents. With this research we know which park characteristics are specifically more or less important for particular subgroups. Therefore, this research can provide a starting point to advice policy makers and urban planners when designing or renovating parks in order to optimize environmental interventions aiming to encourage park visitation and park-based PA. For example, the best strategy to motivate the more active adolescents to remain active is by designing or renovating parks including the presence of a sport field (soccer and basketball). However, to maximize park visitation and park-based PA, it may be essential to target infrastructure and policies that are most likely to reach at-risk subgroups (i.e. those that are currently not visiting or being physically active in parks). Furthermore, these current results indicated that investing in good upkeep and maintenance of parks, and in the provision of a playground or outdoor fitness equipment might be the best strategy among at risk subgroups. Furthermore, the combination of both park characteristics (e.g. a well maintained park with a playground or outdoor equipment) appears to be of great importance. Therefore, future research should investigate whether interaction effects exist between park characteristics, for example which combinations of park characteristics might cause a more beneficial effect or which combinations might create a less beneficial effect for park visitation or park-based PA. Lastly, since the younger inactive adolescents are usually accompanied by family members, urban planners should create public open spaces that attractive for all ages to stimulate more and longer joint park visits and to facilitate their park-based PA [19].

The current study has some limitations that should be taken into account. The most important limitation is that only the preferences for park visitation and park-based PA were studied and not the actual behaviour. Therefore, future natural experiments informed by our findings are warranted to investigate if changes to park characteristics are associated with changes in actual park visitation or actual park-based PA among particular subgroups. Second, in real life, more than ten park characteristics could influence the choice concerning park visitation and park-based PA. Consequently, it is possible that other park characteristics not included here may be important for park visitation or park-based PA for specific subgroups. Nevertheless, the studied park characteristics were carefully selected from previous research [19] and literature [23,25,60–62,72], and the inclusion of more park characteristics would have increased the complexity of the choice experiment and consequently participant burden. Furthermore, by using photographs, some park characteristics were depicted more central than others, which may have influenced participants’ choices. Lastly, the use of computer-generated virtual walkthrough environments [73] could be a suitable solution to accommodate the limitations of the manipulated photographs (i.e. the lack of motion and noise).

A strength of the current study is the use of latent class analysis to investigate whether specific subgroups existed based on similarities in preferences regarding park characteristic for park visitation and park-based PA. Secondly, a large sample of 972 adolescents allowed a sufficient number in each subgroup (i.e. an a priori power analyses was performed in Sawtooth Software, Inc. 2017 [32]). Notwithstanding, for our study both outcomes (i.e. park visitation and park-based PA) yielded similar results, a third major strength was the distinction between the two outcomes because these different behaviours could be hypothesized to be influenced by different park characteristics.

## Conclusions

Using latent class analysis, current results may advise policy makers and urban planners when designing or renovating parks that investing in good upkeep and maintenance of parks, and investing in the provision of a playground or outdoor fitness equipment might be the best strategy to increase both park visitation and park-based PA among at risk subgroups. The presence of a sport field (soccer and basketball) seems to be the best strategy to motivate the more active adolescents to remain active. Future research should investigate which combinations of park characteristics might cause a more/less beneficial effect for park visitation or park-based PA. Urban planners should create public open spaces attractive for all ages to stimulate more and longer joint park visitations to facilitate their park-based PA.

## Supporting information

S1 Additional file Questionnaire Dutch version. (PDF)

S2 Additional file Questionnaire English version. (PDF)

S3 Additional file A detailed overview of the different models for two, three, four and five subgroups is given for park visitation. (PDF)

S4 Additional file A detailed overview of the different models for two, three, four and five subgroups is given for park-based PA. (PDF)

S1 Dataset (SAV)

S1 Table Relative importances of each park characteristic for park visitation, socio-demographics, PA behavior and park use characteristics for the three subgroups identified by latent class analysis (complete table). ^a^ significant difference with subgroup 1; ^b^ significant difference with subgroup 2; ^c^ significant difference with subgroup 3;* p < 0.05.
(PDF)

S2 Table Relative importances of each park characteristic for park-based PA, socio-demographics, PA behavior and park use characteristics for the three subgroups identified by latent class analysis (complete table). ^a^ significant difference with subgroup 1; ^b^ significant difference with subgroup 2; ^c^ significant difference with subgroup 3;* p < 0.05.
(PDF)

## Abbreviations

PA: Physical activity
