# Supplementary material for: Differences in park characteristic preferences for visitation and physical activity among adolescents: A latent class analysis
Source: PLoS One. 2019 Mar 18;14(3):e0212920. doi: 10.1371/journal.pone.0212920 (PMC6422290; doi:10.1371/journal.pone.0212920)
Supplement: S1 Additional file — (PDF) [file pone.0212920.s001.pdf]

S1 Additional file. Questionnaire Dutch version

PARK ONDERZOEK BIJ ADOLESCENTEN

Super dat je wil deelnemen aan dit onderzoek van de Universiteit Gent en de Vrije Universiteit Brussel. We willen onderzoeken welke zaken jongeren belangrijk vinden om een park te bezoeken en er actief te zijn.
Het invullen van de vragenlijst zal ongeveer 30 minuten duren. Het is zeer belangrijk dat je eerlijk antwoordt op alle vragen. Je antwoorden zullen alleen gebruikt worden voor dit onderzoek en anoniem blijven. Als je vragen hebt, mag je deze stellen aan de onderzoeker.

GEEF HIER JE PERSOONLIJKE CODE IN:

DEEL 1: ALGEMENE VRAGEN OVER JEZELF

1. Selecteer jouw school:

2. In welk studiejaar zit je?
☐ 1e middelbaar ☐ 2e middelbaar ☐ 3e middelbaar ☐ 4e middelbaar

3. Welke onderwijsvorm volg je?
☐ 1e graad A stroom
☐ 1e graad B stroom
☐ 2e graad ASO
☐ 2e graad TSO
☐ 2e graad BSO
☐ 2e graad KSO

4. Ik ben … jaar oud
☐ 11 ☐ 12 ☐ 13 ☐ 14 ☐ 15 ☐ 16 ☐ 17 ☐ 18

5. Ik ben een …
☐ jongen ☐ meisje

6. Hoe groot ben je? Noteer je lengte in centimeter.

7. Hoe veel weeg je? Noteer je gewicht in kilogram.

8. Laat je gezondheid het toe om te bewegen (bv: wandelen of tikkertje spelen) en sporten (bv: voetballen).
☐ Nee ☐ Ja

9. Waar ben jij geboren?
☐ België
☐ Andere: ….

10. Waar is je moeder geboren.
☐ België
☐ Andere: ….

11. Waar is je vader geboren.
☐ België
☐ Andere: ….

DEEL 2: EIGEN WOONOMGEVING

12. In welke omgeving woon je? Als je op meerdere plaatsen woont, denk dan aan de plaats waar je het meest tijd doorbrengt. **(1 antwoord aanduiden)**
    ☐ Dorp/gemeente: aan de rand
    ☐ Dorp/gemeente: in het centrum
    ☐ In een stad: aan de rand
    ☐ In een stad: in het centrum

13. Wat is het hoogst behaalde diploma van je vader?
    ☐ lager onderwijs
    ☐ middelbaar onderwijs
    ☐ hoger onderwijs (niet-universitair)
    ☐ universitair onderwijs
    ☐ ik weet het niet/niet van toepassing

14. Wat is het hoogst behaalde diploma van je moeder?
    ☐ lager onderwijs
    ☐ middelbaar onderwijs
    ☐ hoger onderwijs (niet-universitair)
    ☐ universitair onderwijs
    ☐ ik weet het niet/niet van toepassing

15. SES: family affluence scale
    - Is jouw gezin in het bezit van een auto?
      ☐ Nee ☐ Ja: 1 ☐ Ja: 2 of meer
    - Heb je een eigen kamer, alleen voor jezelf?
      ☐ Nee ☐ Ja
    - Hoe vaak ging je met je gezin op vakantie naar het buitenland tijdens de voorbije zes maanden?
      ☐ Niet ☐ Eén keer ☐ Twee keer ☐ Meer dan twee keer
    - Hoeveel computers hebben jullie in je gezin (laptops tellen mee, smartphones en computerconsoles niet)?
      ☐ Geen ☐ Eén ☐ Twee ☐ Meer dan twee
    - Hoeveel badkamers (kamer met bad en/of douche) hebben jullie thuis?
      ☐ Geen ☐ Eén ☐ Twee ☐ Meer dan twee
    - Hebben jullie thuis een vaatwasmachine?
      ☐ Nee ☐ Ja

DEEL 3: GEBRUIK VAN PARKEN

16. Hoe vaak heb je een park bezocht in de voorbije drie maanden? **(1 antwoord aanduiden)**
    ☐ Dagelijks
    ☐ 2-3 keer per week
    ☐ Eén keer per week
    ☐ 2-3 keer per maand
    ☐ Eén keer per maand
    ☐ Minder dan één keer per maand
    ☐ Ik heb geen enkel park bezocht in de laatste 3 maanden (Ga naar vraag …)

17. Hoe lang bleef je meestal in het park bij een bezoek in de voorbije drie maanden? **(1 antwoord aanduiden)**
    ☐ <30 minuten
    ☐ 30-59 minuten

- ☐ 1 tot 2 uur
- ☐ 2 tot 3 uur
- ☐ 3 tot 4 uur
- ☐ 4 of meer uren

18. Wanneer je in de voorbije drie maanden naar het park ging, met wie ging je? **(Selecteer alle antwoorden die van toepassing zijn)**
    - ☐ Alleen
    - ☐ (Groot)Ouders, tantes of nonkels
    - ☐ Broer(s) of zus(sen), nichten of neven
    - ☐ vrienden
    - ☐ georganiseerde groep (bv: scouts)
    - ☐ Hond
    - ☐ Andere: ________________________________

19. Welke activiteiten deed je meestal wanneer je in de voorbije 3 maanden naar het park ging? (vb: voetballen, wandelen) **(meerdere antwoorden mogelijk)**
    - ☐ Zitten/liggen
    - ☐ Staan
    - ☐ Wandelen
    - ☐ Fietsen
    - ☐ Skaten/BMX/Rollerskaten
    - ☐ Balsport
    - ☐ Tik- en loopspelen
    - ☐ Joggen
    - ☐ Yoga
    - ☐ Lichaamsoefeningen (bv: fitness)
    - ☐ Andere: _________________________

20. Welk transportmiddel gebruikte je meestal wanneer je in de voorbije 3 maanden naar het park ging? **(meerdere antwoorden mogelijk)**
    - ☐ Te voet
    - ☐ Met de fiets/skateboard/step/logboard/…
    - ☐ Met de auto, motor of brommer (als passagier)
    - ☐ Met het openbaar vervoer (trein, bus, tram, metro)

21. Hoe lang duurt het om van bij je thuis tot bij het dichtstbijzijnde park te wandelen? **(1 antwoord aanduiden)**
    - ☐ 1-5 min
    - ☐ 6-10 min
    - ☐ 11-20 min
    - ☐ 21-30 min
    - ☐ meer dan 30 min
    - ☐ Weet ik niet

22. Hoe lang duurt het om van bij je thuis tot bij het park te wandelen dat je het meeste bezoekt? **(1 antwoord aanduiden)**
    - ☐ 1-5 min
    - ☐ 6-10 min
    - ☐ 11-20 min
    - ☐ 21-30 min
    - ☐ meer dan 30 min
    - ☐ Weet ik niet/ ik ga nooit naar een park

DEEL 4: FYSIEKE ACTIVITEIT

In het volgende deel willen we wat meer weten over de lichamelijke activiteit die jij doet in het dagelijkse leven. Met lichamelijke activiteit bedoelen we alle activiteiten behalve liggen, zitten en stilstaan, dus sporten zoals voetbal en zwemmen maar ook wandelen met de hond of fietsen. De vragen gaan over jouw lichamelijke activiteit **gedurende de afgelopen 7 dagen**. Beantwoordt alsjeblieft alle vragen, ook al vind je jezelf niet zo lichamelijk actief. Denk aan activiteiten die je doet op school, in en rond het huis, om van de ene naar de andere plaats te komen en activiteiten in je vrije tijd voor recreatie, training of sport.

Denk aan alle **zware** lichamelijke activiteiten die je deed in **de afgelopen 7 dagen**. **Zware** lichamelijke activiteiten zijn activiteiten die veel lichamelijke inspanning kosten en voor een veel snellere ademhaling zorgen. Denk *alleen* aan de activiteiten die je ten minste 10 minuten per keer hebt gedaan.

1. Als je denkt aan **de afgelopen 7 dagen**, op hoeveel van deze dagen heb je **zware** lichamelijke activiteiten gedaan zoals zware lasten tillen, spitten, aerobics of wielrennen?

_ **dagen per week**

☐ geen zware lichamelijke activiteiten → **ga naar vraag 3**

2. Op de dagen waarop je zwaar lichamelijk actief was, hoeveel tijd heb je daar dan gewoonlijk aan besteed?

_ **minuten per dag**

☐ Weet niet / niet zeker

Denk aan activiteiten die **matige** lichamelijke inspanning kosten en die je in **de afgelopen 7 dagen** hebt gedaan. **Matig** intensieve lichamelijke activiteit laat je iets sneller ademen dan normaal. Denk weer alleen aan activiteiten die je ten minste 10 minuten per keer hebt gedaan.

3. Als je denkt aan **de afgelopen 7 dagen**, op hoeveel van deze dagen heb je **matig** intensieve lichamelijke activiteit verricht, zoals het dragen van lichte lasten, fietsen in een normaal tempo of dubbeltennis? Laat wandelen hier buiten beschouwing.

_ **dagen per week**

☐ geen matig lichamelijke activiteiten → **ga naar vraag 5**

4. Op de dagen waarop je **matig** intensief lichamelijk actief was, hoeveel tijd heb je daar dan gewoonlijk aan besteed?

_ **minuten per dag**

☐ Weet niet / niet zeker

5. Als je denkt aan **de afgelopen 7 dagen**, op hoeveel dagen heb je tenminste 10 minuten per keer **gewandeld**? Denk hierbij aan wandelen op school en thuis, wandelen om van de ene naar de andere plaats te komen, en al het andere wandelen dat je deed tijdens recreatie, sport of vrijetijdsbesteding.

_ **dagen per week**

☐ geen wandelen → **ga naar vraag 7**

6. Op de dagen waarop je ten minste 10 minuten per keer **wandelde**, hoeveel tijd heb je daar dan gewoonlijk aan besteed?

_ **minuten per dag**

☐ Weet niet / niet zeker

7. Hoeveel tijd bracht je gewoonlijk **zittend** door gedurende een **doordeweekse** dag **in de afgelopen 7 dagen**? Bij deze tijd mag zitten achter een bureau, tijd die zittend wordt doorgebracht met vrienden, zittend lezen, studeren of tv kijken worden gerekend.

_ **minuten per dag**

☐ Weet niet / niet zeker

8. Ben je lid van een sport club of fitness centrum?

☐ Nee ☐ Ja

9. Welke sport beoefen je in deze sport club of fitness centrum?

☐ voetbal/basket
☐ fitness
☐ joggen/lopen
☐ fietsen
☐ Skaten/BMX/Rollerskaten
☐ wandelen
☐ zwemmen
☐ tennis
☐ badminton
☐ turnen
☐ Andere:

KEUZETAAK 1

Beeld je in dat je **overdag in het weekend naar een park gaat.** Het is mooi weer, niet te warm en niet te koud en het regent niet. Je zal telkens 2 parken te zien krijgen op een foto. Je kent beide parken goed en ze zijn beide vlakbij je huis Het is de bedoeling dat je het park uitkiest dat je het **meest uitnodigt om naartoe te gaan.** Neem genoeg tijd om beide foto's goed te bekijken.

In totaal krijg je 10 situaties te zien waarbij je telkens moet aanduiden welk park je verkiest om naartoe te gaan.

KEUZETAAK 2

Beeld je in dat je **overdag in het weekend in een park bent**. Het is mooi weer, niet te warm en niet te koud en het regent niet. Je zal telkens 2 parken te zien krijgen op een foto. Je kent beide parken goed en ze zijn beide vlakbij je huis. Het is de bedoeling dat je het park uitkiest dat je het meest uitnodigt om te bewegen. Met bewegen bedoelen we alle activiteiten behalve zitten en liggen, zoals: spelen, wandelen (met de hond), sporten (vb: voetbal), ... Neem genoeg tijd om beide foto's goed te bekijken.

Na elke keuzetaak zal je kunnen aanduiden of je in het park dat je koos ook echt zou bewegen. Als je in het gekozen park dus iets anders zou doen (zoals bijvoorbeeld al zittend picknicken of op het gras liggen), of verkiest niet in dit park te blijven, kan je dat aanduiden.

Er is geen juiste of foute oplossing, we zijn enkel geïnteresseerd in wat jij belangrijk vindt in het park.
In totaal krijg je 10 situaties te zien waarbij je telkens moet aanduiden welk park je verkiest om actief te zijn.

23. Waren de vragen met de foto's die je juist beantwoord hebt toepasselijk voor jou? Je kan je opmerkingen hier noteren.
24. Heb je nog opmerkingen over je eigen bewegingsgedrag en/of de foto's/situaties die je gezien hebt?

DEEL 6: SOCIALE OMGEVING

25. Hoe vaak moedigen je vrienden, gezin en klasgenoten je aan om te bewegen?

	Nooit	Zelden	Soms	Vaak	Zeer vaak	Heb ik niet/niet van toepassing
vrienden	○	○	○	○	○	○
gezin	○	○	○	○	○	○
klasgenoten	○	○	○	○	○	○

26. Duid aan in welke mate u akkoord gaat met volgende stellingen.

	Helemaal niet akkoord	Eerder niet akkoord	Soms niet/soms wel akkoord	Eerder akkoord	Helemaal akkoord	Heb ik niet/niet van toepassing
Mijn vrienden vinden dat ik op de meeste dagen van de week in mijn vrije tijd zou moeten bewegen	○	○	○	○	○	○
Mijn broers/zussen vinden dat ik op de	○	○	○	○	○	○

meeste dagen van de week in mijn vrije tijd zou moeten bewegen						
Mijn ouders vinden dat ik op de meeste dagen van de week in mijn vrije tijd zou moeten bewegen	○	○	○	○	○	○
Mijn klasgenoten vinden dat ik op de meeste dagen van de week in mijn vrije tijd zou moeten bewegen	○	○	○	○	○	○

27. Hoe vaak bewegen vrienden, gezin en klasgenoten in hun vrije tijd?

	Nooit of één keer per jaar	Enkele keren per jaar	Verschillende keren per maand	Verschillende keren per week	Bijna dagelijks	Heb ik niet/niet van toepassing
vrienden	○	○	○	○	○	○
Broers/zussen	○	○	○	○	○	○
ouders	○	○	○	○	○	○
klasgenoten	○	○	○	○	○	○

28. Duid aan in welke mate u akkoord gaat met volgende stellingen.

	Helemaal niet akkoord	Eerder niet akkoord	Soms niet/soms wel akkoord	Eerder akkoord	Helemaal akkoord	Heb ik niet/niet van toepassing
Ik hecht belang aan de mening van mijn vrienden omtrent hoe vaak ik moet bewegen.	○	○	○	○	○	○
Ik hecht belang aan de mening van mijn gezin omtrent hoe vaak ik moet bewegen.	○	○	○	○	○	○
Ik hecht belang aan de mening van mijn klasgenoten omtrent hoe vaak ik moet bewegen	○	○	○	○	○	○

29. Hoe vaak vragen vrienden, gezin en klasgenoten om samen te bewegen?

	Helemaal niet akkoord	Eerder niet akkoord	Soms niet/soms wel akkoord	Eerder akkoord	Helemaal akkoord	Heb ik niet/niet van toepassing
vrienden	○	○	○	○	○	○

Broers/zussen	○	○	○	○	○	○
ouders	○	○	○	○	○	○
klasgenoten	○	○	○	○	○	○

30. Ik heb _________________ beste vrienden
