# Supplementary material for: Differences in park characteristic preferences for visitation and physical activity among adolescents: A latent class analysis
Source: PLoS One. 2019 Mar 18;14(3):e0212920. doi: 10.1371/journal.pone.0212920 (PMC6422290; doi:10.1371/journal.pone.0212920)
Supplement: S2 Additional file — (PDF) [file pone.0212920.s002.pdf]

S2 Additional file. Questionnaire English version

QUESTIONNAIRE PARK RESEARCH AMONG ADOLESCENTS

Thank you for participating in this research from the Ghent University and the Vrije Universiteit Brussel.
We want to investigate which characteristics adolescents consider important to visit a park and to be physically active in the park.

Completing the questionnaire will take about 30 minutes. It is very important that you answer honestly to all questions. Your answers will only be used for this research and remain anonymous. If you have questions, you can ask them to the researcher.

ENTER YOUR PERSONAL CODE HERE:

PART 1: GENERAL INFORMATION

1. What is the name of your school:

2. Which grade in school are you follow?
☐ 1e secondary ☐ 2e secondary ☐ 3e secondary ☐ 4e secondary

3. What type of education do you follow?
☐ 1e grade A
☐ 1e grade B
☐ 2e grade ASO
☐ 2e grade TSO
☐ 2e grade BSO
☐ 2e grade KSO

4. How old are you now?
☐ 11 ☐ 12 ☐ 13 ☐ 14 ☐ 15 ☐ 16 ☐ 17 ☐ 18

5. Are you a girl or a boy?
☐ boy ☐ girl

6. What is your height? …. cm

7. What is your current weight? …. kg

8. Let your health allows it to exercise (eg walking or playing tag) and sports (eg football)?
☐ No ☐ Yes

9. Where are you born?
☐ Belgium
☐ Other: ….

10. Where is your mother born?
☐ Belgium
☐ Andere: ….

11. Where is your father born?
☐ Belgium

☐ Andere: ….

PART 2: YOUR NEIGHBORHOOD

12. In which neighborhood do you live? If you live in several places, think of the place where you spend most of your time. (Indicate one answer)?
    - Village / town: at the edge
    - Village / municipality: in the center
    - In a city: at the edge
    - In a city: in the center

13. What is the highest level of education of your father?
    - primary education
    - secondary education
    - higher education (non-university)
    - university education
    - I do not know / do not apply

14. What is the highest level of education of your mother?
    - primary education
    - secondary education
    - higher education (non-university)
    - university education
    - I do not know / do not apply

15. SES: familiy affluence scale
    - Does your family own a car?
      ☐ No ☐ Yes: 1 ☐ Yes: 2 or more
    - Do you have a private room, only for yourself?
      ☐ No ☐ Yes
    - How often did you go on holiday with your family during the past six months?
      ☐ Non ☐ ones ☐ Twice ☐ More than twice
    - How many computers do you have in your family (laptops count, smartphones and computer consoles not)?
      ☐ Non ☐ one ☐ Two ☐ More than two
    - How many bathrooms (room with bath and / or shower) do you have at home?
      ☐ Non ☐ one ☐ Two ☐ More than two
    - Do you have a dishwasher at home?
      ☐ No ☐ Yes:

PART 3: PARK USE

16. How often have you visited a park in the past three months? (Indicate one answer)
    - Daily
    - 2-3 times a week
    - Once a week
    - 2-3 times a month
    - Once a month
    - Less than once a month

- I have not visited any park in the last 3 months (Go to question ...)

17. How long did you usually stay in the park during a visit in the past three months? (Indicate one answer)
    - <30 minutes
    - 30-59 minutes
    - 1 tot 2 hours
    - 2 tot 3 hours
    - 3 tot 4 hours
    - 4 of meer hours

18. When you went to the park in the past three months, who were you traveling with? (Select all the answers that apply)
    - Alone
    - (Grant) Parents, aunts or uncles
    - Brother (s) or sister (s), nieces or cousins
    - friends
    - organized group (eg: scouts)
    - Dog
    - Other: ________________________________

19. What activities did you usually do when you went to the park in the past 3 months? (eg: football, walking) (multiple answers possible)
    - Sitting / lying down
    - Stand
    - Hiking
    - Bicycles
    - Skating / BMX / Rollerskating
    - Ball sport
    - Tap and run games
    - To jog
    - Yoga
    - Body exercises (eg: fitness)
    - Other: _________________________

20. Which means of transport did you usually use when you went to the park in the past 3 months? (multiple answers possible)
    - By foot
    - By bike / skateboard / scooter / logboard / ...
    - By car, motorcycle or moped (as a passenger)
    - By public transport (train, bus, tram, metro)

21. How long does it take to walk from your home to the nearest park? (Indicate one answer)
    - 1-5 minutes
    - 6-10 minutes
    - 11-20 minutes
    - 21-30 minutes
    - more than 30 minutes
    - I don't know

22. How long does it take to walk from your home to the park that you visit the most? (Indicate one answer)
    - 1-5 minutes
    - 6-10 minutes
    - 11-20 minutes
    - 21-30 minutes
    - more than 30 minutes

- I don’t know/ I never go to a park

PART 4: PHYSICAL ACTIVITY

We are interested in finding out about the kinds of physical activities that people do as part of their everyday lives. The questions will ask you about the time you spent being physically active in **the last 7 days**. Please answer each question even if you do not consider yourself to be an active person. Please think about the activities you do at work, as part of your house and yard work, to get from place to place, and in your spare time for recreation, exercise or sport.

Think about all the **vigorous** activities that you did in the **last 7 days**. Vigorous physical activities refer to activities that take hard physical effort and make you breathe much harder than normal. Think only about those physical activities that you did for at least 10 minutes at a time.

1. During the last 7 days, on how many days did you do vigorous physical activities like heavy lifting, digging, aerobics, or fast bicycling?

_ **days per week**

☐ No vigorous physical activities → Skip to question 3

2. How much time did you usually spend doing vigorous physical activities on one of those days?

_ **minutes per day**

☐ Don’t know/Not sure

Think about all the **moderate** activities that you did in **the last 7 days**. Moderate activities refer to activities that take moderate physical effort and make you breathe somewhat harder than normal. Think only about those physical activities that you did for at least 10 minutes at a time..

3. During the last 7 days, on how many days did you do moderate physical activities like carrying light loads, bicycling at a regular pace, or doubles tennis? Do not include walking.

_ **days per week**

☐ No moderate physical activities → Skip to question 5

4. How much time did you usually spend doing moderate physical activities on one of those days?

_ **minutes per day**

☐ Don’t know/Not sure

5. Think about the time you spent **walking** in the **last 7 days.** This includes at work and at home, walking to travel from place to place, and any other walking that you have done solely for recreation, sport, exercise, or leisure.

_ **days per week**

☐ No walking → Skip to question 7

6. How much time did you usually spend walking on one of those days?

_ **minutes per day**

☐ Don’t know/Not sure

7. The last question is about the time you spent **sitting** on weekdays during the **last 7 days**. Include time spent at work, at home, while doing course work and during leisure time. This may include time spent sitting at a desk, visiting friends, reading, or sitting or lying down to watch television.

_ **minutes per day**

☐ Don’t know/Not sure

8. Are you a member of a sports club or fitness center?

☐ No ☐ Yes

9. What sport do you practice in this sports club or fitness center?

- football / basket
- fitness
- jogging / running
- bicycles
- Skating / BMX / Rollerskating
- hiking
- swimming
- tennis
- badminton
- gymnastics
- Other:

CHOICETASK 1

Imagine that you are **going to a park during the day on the weekend**. The weather is ideal, it is not too warm, not too cold, there is no wind, and it is not raining. You will see two photographs of a park. Imagine that you know both parks and they are both close to your home. Please take your time to look at the photographs, and then select the park that is **most inviting to visit**.

In total, you will be presented with 10 tasks where you have to choose the park you prefer to visit. There is no right or wrong solution, we are just interested in what you consider to be the most important characteristics in a park.

CHOICETASK 2

Imagine that you are **going to a park during the day on the weekend**. The weather is ideal, it is not too warm, not too cold, there is no wind, and it is not raining. You will see two photographs of a park. Imagine that you know both parks and they are both close to your home. Please take your time to look at the photographs, and then select the park that was **most inviting to be physically active**. By physical activity we mean: "all activities except sitting and lying down, such as playing active games, walking the dog or sports such as soccer".

After each choice task (where you chose the park they preferred for physical activity), you will be asked if you would also be actually active in that park.

In total, you will be presented with 10 tasks where you have to choose the park you prefer to visit. There is no right or wrong solution, we are just interested in what you consider to be the most important characteristics in a park.

23. Were the questions with the photos that you answered correctly applicable to you? You can note your comments here.
24. Do you have any comments about your own physcial activity behavior and / or the photos / situations you have seen?

PART 6: SOCIAL ENVIRONMENT

25. How often do your friends, family and classmates encourage you to move?

	Never	Rarely	Sometimes	Often	Very often	I do not have/not applicable
friends	○	○	○	○	○	○
family	○	○	○	○	○	○
classmates	○	○	○	○	○	○

26. Indicate to what extent you agree with the following statements.

	Strongly Disagree	Disagree	Undecided	Agree	Strongly Agree	I do not have/not applicable
My friends think I should be physically active in my spare time on most days of the week	○	○	○	○	○	○
My brothers / sisters believe that I should be physically	○	○	○	○	○	○

active in my spare time on most days of the week						
My parents think I should be physically active in my spare time on most days of the week	○	○	○	○	○	○
My classmates think I should be physically active in my spare time on most days of the week	○	○	○	○	○	○

27. How often are friends, family and classmates be physically active in their spare time?

	Never or once a year	Several times a year	Several times a month	Several times a week	Almost daily	I do not have/not applicable
friends	○	○	○	○	○	○
brothers / sisters	○	○	○	○	○	○
parents	○	○	○	○	○	○
classmates	○	○	○	○	○	○

28. Indicate to what extent you agree with the following statements.

	Strongly Disagree	Disagree	Undecided	Agree	Strongly Agree	I do not have/not applicable
I attach importance to the opinion of my friends about how often I have to be physically active.	○	○	○	○	○	○
I attach importance to the opinion of my family about how often I have to be physically active.	○	○	○	○	○	○
I attach importance to the opinion of my classmates about how often I have to be physically active	○	○	○	○	○	○

29. How often do friends, family and classmates ask to be physically active together?

	Strongly Disagree	Disagree	Undecided	Agree	Strongly Agree	I do not have/not applicable
friends	○	○	○	○	○	○
brothers / sisters	○	○	○	○	○	○
parents	○	○	○	○	○	○
classmates	○	○	○	○	○	○

30. I have_________________ best friends
