# Supplementary material for: Differences in park characteristic preferences for visitation and physical activity among adolescents: A latent class analysis
Source: PLoS One. 2019 Mar 18;14(3):e0212920. doi: 10.1371/journal.pone.0212920 (PMC6422290; doi:10.1371/journal.pone.0212920)
Supplement: S3 Additional file — (PDF) [file pone.0212920.s003.pdf]

S3 Additional file. A detailed overview of the different models for two, three, four and five subgroups is given for park visitation.

Table S3.1. Latent Class Estimation for park visitation

Number of replications	5
Maximum number of iterations	100
Convergence limit for log likelihood	0,01000
Random number seed	1
Null log-likelihood	-5384,36730

Table S3.2. Summary of best replications for park visitation

Groups	Log-likelihood	Pct Cert	AIC	CAIC	BIC	ABIC	Chi-Square	Relative Chi-Square
2	-3531	34.43	7123	7370	7339	7241	3707	120
3	-3454	35.86	7002	7376	7329	7179	3861	82
4	-3410	36.66	6947	7448	7385	7185	3948	62
5	-3388	37.07	6934	7562	7483	7232	3992	51

**Pct Cert= Percent Certainty; AIC= Akaike Info Criterion; CAIC= Consistent Akaike Info Criterion; BIC= Bayesian Information Criterion; ABIC= Adjusted Bayesian Info Criterion*

Table S3.3. Tabulation with the comparison between different groups for park visitation

Tabulation of 2 group vs. 3 group solutions

	1	2	3	Total
1	665	10	141	816
2	1	137	17	155
Total	666	147	158	971

Tabulation of 3 group vs. 4 group solutions

	1	2	3	4	Total
1	615	3	44	4	666
2	1	133	13	0	147
3	17	4	85	52	158
Total	633	140	142	56	971

Tabulation of 4 group vs. 5 group solutions

	1	2	3	4	5	Total
1	12	492	1	120	8	633
2	0	0	1	2	137	140
3	124	1	0	15	2	142
4	10	8	36	0	2	56
Total	146	501	38	137	149	971

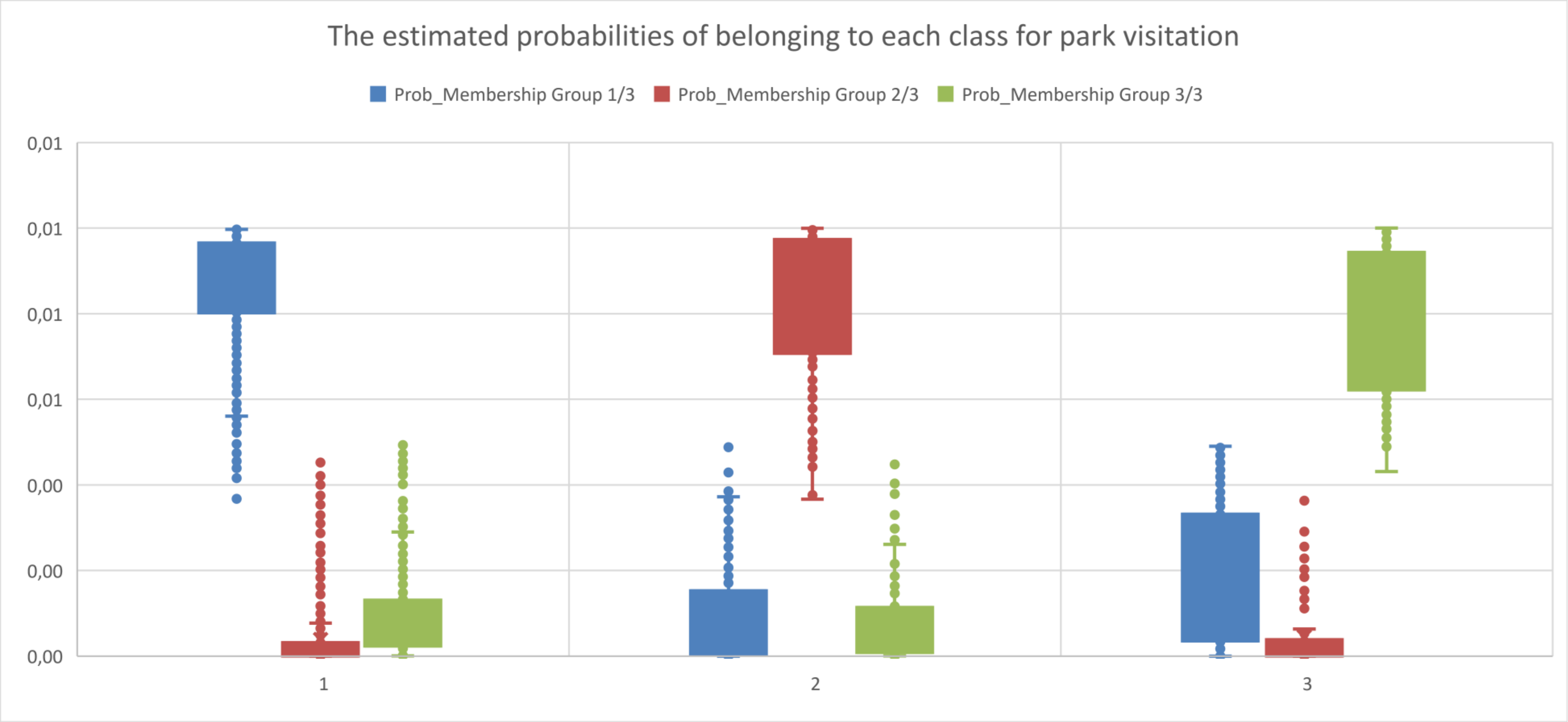

Figure S3.1. The estimated probabilities of belonging to each class of the 3-class model for park visitation
