# Supplementary material for: Differences in park characteristic preferences for visitation and physical activity among adolescents: A latent class analysis
Source: PLoS One. 2019 Mar 18;14(3):e0212920. doi: 10.1371/journal.pone.0212920 (PMC6422290; doi:10.1371/journal.pone.0212920)
Supplement: S4 Additional file — (PDF) [file pone.0212920.s004.pdf]

S4 Additional file. A detailed overview of the different models for two, three, four and five subgroups is given for park-based PA.

Table S4.1. Latent Class Estimation for park-based PA

Number of replications	5
Maximum number of iterations	100
Convergence limit for log likelihood	0,01000
Random number seed	1
Null log-likelihood	-9722,85835

Table S4.2. Summary of best replications for park-based PA

Groups	Log-likelihood	Pct Cert*	AIC*	CAIC*	BIC*	ABIC*	Chi-Square	Relative Chi-Square
2	-7417	23.72	14900	15169	15136	15031	4612	140
3	-7223	25.71	14547	14955	14905	14746	4999	100
4	-7089	27.09	14312	14859	14792	14579	5267	79
5	-6995	28.06	14158	14843	14759	14492	5456	65

**Pct Cert= Percent Certainty; AIC= Akaike Info Criterion; CAIC= Consistent Akaike Info Criterion; BIC= Bayesian Information Criterion; ABIC= Adjusted Bayesian Info Criterion*

Table S4.3. Tabulation with the comparison between different groups for park-based PA

Tabulation of 2 group vs. 3 group solutions

	1	2	3	Total
1	152	4	411	567
2	1	337	65	403
Total	153	341	476	970

Tabulation of 3 group vs. 4 group solutions

	1	2	3	4	Total
1	4	1	148	0	153
2	303	0	0	38	341
3	129	336	11	0	476
Total	436	337	159	38	970

Tabulation of 4 group vs. 5 group solutions

	1	2	3	4	5	Total
1	0	0	414	4	18	436
2	6	0	22	217	92	337
3	151	0	1	0	7	159
4	0	38	0	0	0	38
Total	157	38	437	221	117	970

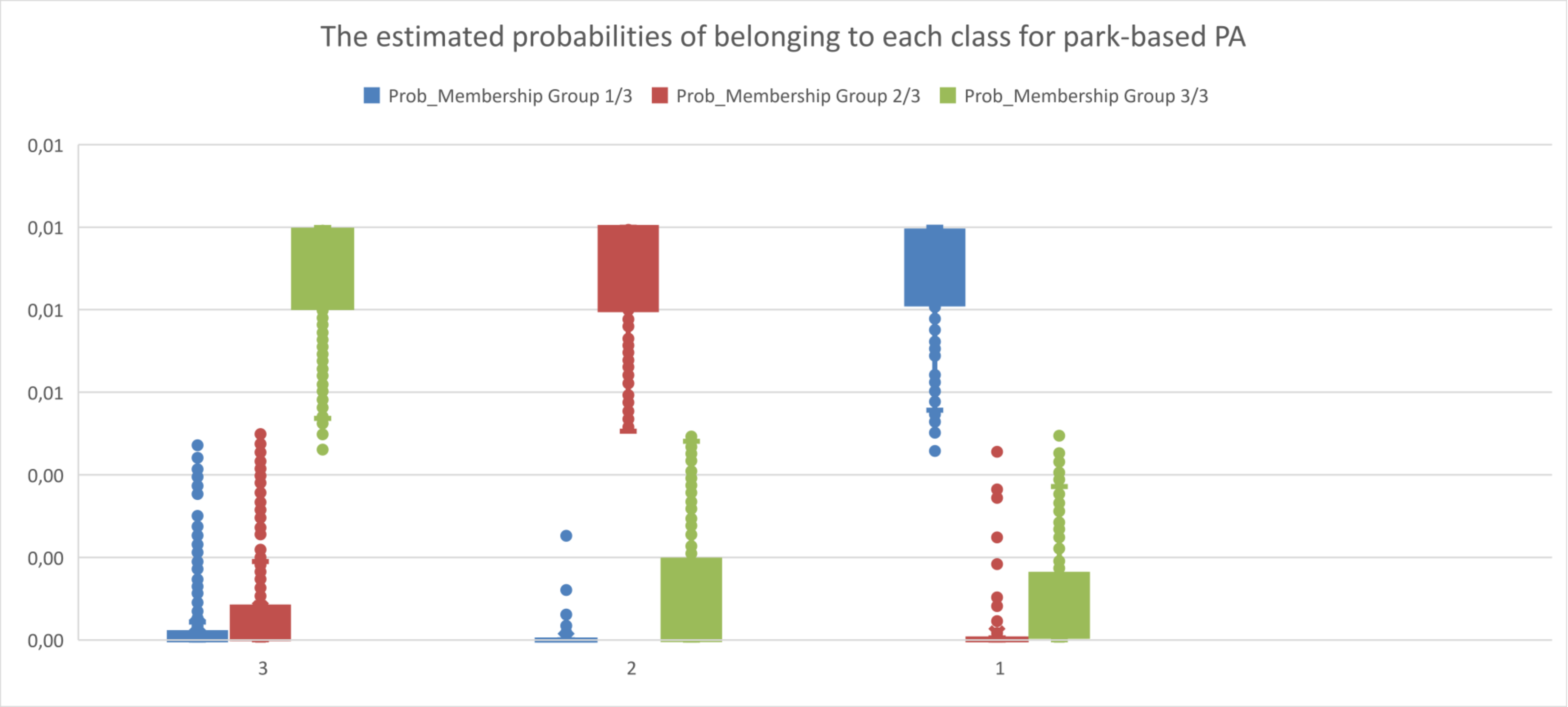

Figure S4.1. The estimated probabilities of belonging to each class of the 3-class model for park-based PA
