# Supplementary material for: Differences in park characteristic preferences for visitation and physical activity among adolescents: A latent class analysis
Source: PLoS One. 2019 Mar 18;14(3):e0212920. doi: 10.1371/journal.pone.0212920 (PMC6422290; doi:10.1371/journal.pone.0212920)
Supplement: S1 Table — a significant difference with subgroup 1; b significant difference with subgroup 2; c significant difference with subgroup 3;* p < 0.05. (PDF) [file pone.0212920.s006.pdf]

S1 Table. Relative importances of each park characteristic for park visitation, socio-demographics, PA behavior and park use characteristics for the three subgroups identified by latent class analysis (complete table)

S1 Table. Relative importances of each park characteristic for park visitation, socio-demographics, PA behavior and park use characteristics for the three subgroups identified by latent class analysis (complete table).

	Subgroup 1	Subgroup 2	Subgroup 3	*p-value*
Subgroup Sizes (n)	n= 666	n=147	n=158	
	68.6%	15.1%	16.3%	
Relative Importances (M (95 % CI))				
Upkeep	48.1 (47.5,48.6)	25.7 (24.1,27.2)	26.2 (24.6,27.8)	
Playground/outdoor fitness	15.3 (14.9,15.8)	14.6 (13.5,15.6)	21.1 (19.7,22,6)	
Sport field	8.0 (7.6,8.4)	30.2 (28.8,31.6)	9.3 (8.4,10.3)	
Activity peers	5.0 (4.7,5.2)	6.5 (5.8,7.2)	11.3 (10.2,12.3)	
Homeless person	5.6 (5.4,5.9)	4.3 (3.7,4.8)	8.2 (7.2,9.3)	
Walking paths	5.4 (5.2,5.6)	6.1 (5.7,6.6)	6.9 (6.3,7.5)	
Naturalness	4.5 (4.3,4.7)	4.9 (4.3,5.4)	6.3 (5.6,7.1)	
Mother with a child	3.4 (3.3,3.6)	2.6 (2.3,3.0)	3.4 (3.0,3.8)	
Benches	2.4 (2.2,2.5)	2.8 (2.5,3.2)	4.1 (3.6,4.6)	
Drinking fountain	2.2 (2.1,2.4)	2.4 (2.1,2.7)	3.1 (2.7,3.5)	
Socio-demographic characteristics				
Age (years, M±SD)	13.2 ± 1.2 [b]	13.5 ± 1.4 [a]	13.4 ± 1.3	0.015*
Gender (% women)	61.6 [b]	20.4 [a,c]	53.8 [b]	<0.001*
Born in Belgium (%)	93.7	95.9	93.7	0.576
Other ethnicity (%)	31.1 [b,c]	44.2 [a]	46.8 [a]	<0.001*
Education (% at least one parent high educated)	79.1 [c]	81.4 [c]	66.7 [a,b]	0.011*
SES (%)				0.486
- Low	4.8	5.4	7.6	
- Medium	30.2	30.6	34.2	
- High	65.0	63.9	58.2	
Living area (%)				0.007*
- Rural	11.1	7.5	10.8	
- Suburban	60.5	49.7	52.5	
- Urban	28.4	42.9	36.7	
Meets PA guidelines (%)	43.1	58.5	44.9	0.003*
Member of sport club (%)	67.7	79.6	57.6	<0.001*
How many friends do you have?	6.8 ± 9.1	11.6 ± 15.3	8.9 ± 12.3	<0.001*
Categories z-scores (BMI) (%)				0.374
- Underweight	6.7	2.8	6.0	
- Normal weight	86.7	90.1	84.8	
- Overweight	6.7	7.1	9.3	

PA behavior (min/week)				
Light PA (M±SD)	248.3 ± 211.4	236.6 ± 217.2	267.3 ± 239.3	0.448
Moderate-to-vigorous intensity PA (M±SD)	438.6 ± 367.2 [b]	569.3 ± 390.0 [a,c]	443.7 ± 382.0 [b]	0.001*
Park use characteristics				
Frequency of visitation (M±SD)	3.5 ± 1.7	3.8 ± 1.9	3.8 ±1.8	0.032*
Park duration (min/3 months) (M±SD)	75.3 ± 57.1 [b]	96.3 ± 68.3 [a]	85.2 ± 64.4	0.001*
Walking distance to closest park (min) (M±SD)	14.0 ± 12.3	12.2 ± 11.8	11.7 ± 11.0	0.058
Accompaniment to parks				
Friends (%)	60.5	76.7	61.0	0.002*
Parents/grandparents/aunt/uncle (%)	38.6	14.7	29.1	<0.001*
Dog (%)	16.0	7.0	7.8	0.003*
(Step)brother/sister/niece/nephew (%)	37.9	27.9	34.8	0.096
Organised group (%)	19.3	17.8	13.5	0.272
Alone (%)	14.6	14.7	14.2	0.990
Usual activities during park visitation				
Walking (%)	67.8	34.1	53.2	<0.001*
Ball sports (%)	37.7	72.9	39.7	<0.001*
Sitting/lying down (%)	39.8	27.9	34.0	0.029*
Biking (%)	31.8	27.1	26.2	0.309
Jogging (%)	21.6	17.1	19.1	0.473
Standing (%)	16.0	11.6	20.6	0.135
Skating (%)	14.1	18.6	14.2	0.416
Active games (%)	15.7	9.3	15.6	0.173
Exercising (%)	10.4	17.8	10.6	0.056
Yoga (%)	1.0	0.8	0.7	0.913
Usual transportation to parks in the last three months				
Walking (%)	69.7	70.5	73.8	0.644
Biking (%)	40.9	41.1	34.8	0.395
Car/moped/motorbike as a passenger (%)	27.0	20.9	20.6	0.150
Public transportation (%)	17.0	16.3	17.7	0.951
Skateboard/long board/scooter (%)	10.8	11.6	9.2	0.801

[a] significant difference with subgroup 1; [b] significant difference with subgroup 2; [c] significant difference with subgroup 3;* $p < 0.05$
