# Supplementary material for: Differences in park characteristic preferences for visitation and physical activity among adolescents: A latent class analysis
Source: PLoS One. 2019 Mar 18;14(3):e0212920. doi: 10.1371/journal.pone.0212920 (PMC6422290; doi:10.1371/journal.pone.0212920)
Supplement: S2 Table — a significant difference with subgroup 1; b significant difference with subgroup 2; c significant difference with subgroup 3;* p < 0.05. (PDF) [file pone.0212920.s007.pdf]

S2 Table. Relative importances of each park characteristic for park-based PA, socio-demographics, PA behavior and park use characteristics for the three subgroups identified by latent class analysis (complete table)

S2 Table. Relative importances of each park characteristic for park-based PA, socio-demographics, PA behavior and park use characteristics for the three subgroups identified by latent class analysis (complete table).

	Subgroup 1	Subgroup 2	Subgroup 3	*p-value*
Subgroups Sizes (n)	n=341	n= 153	n=476	
	35.2%	15.8%	49.1%	
Average relative importances % (M (95% CI))				
Upkeep (M (95% CI))	44.0 (42.6,45.4)	32.3 (30.3,34.2)	41.9 (40.8,43.0)	
Playground/outdoor fitness	14.5 (13.9,15.2)	15.3 (14.3,16.3)	17.6 (17.0,18.3)	
Sport field	9.1 (8.2,9.9)	23.1 (21.2,25.0)	9.7 (7.0,10.3)	
Activity peers	6.3 (5.8,6.9)	6.0 (5.3,6.7)	6.2 (5.8,6.7)	
Homeless person	6.5 (6.0,7.0)	5.0 (4.4,5.5)	5.7 (5.3,6.0)	
Walking paths	5.8 (5.5,6.1)	5.8 (5.4,6.3)	5.7 (5.5,5.9)	
Naturalness	4.9 (4.6,5.3)	4.8 (4.3,5.4)	4.8 (4.5,5.1)	
Mother with a child	3.4 (3.2,3.7)	2.8 (2.5,3.1)	3.4 (3.2,3.6)	
Benches	2.9 (2.7,3.2)	2.6 (2.2,2.9)	2.6 (2.4,2.8)	
Drinking fountain	2.4 (2.2,2.6)	2.4 (2.1,2.7)	2.4 (2.3,2.6)	
Socio-demographic characteristics				
Age (years, M±SD)	13.6 ± 1.2 [c]	13.5 ± 1.4 [c]	13.1 ± 1.2 [a,b]	<0.001*
Gender (% women)	65.1 [b,c]	19.6 [a,c]	57.1 [a,b]	<0.001*
Born in Belgium (%)	93.8	95.4	93.7	0.725
Other ethnicity (%)	38.1	35.9	33.8	0.448
Education (% at least one parent high educated)	74.3	82.1	78.4	0.223
SES (%)				0.242
- Low	6.5	5.2	4.6	
- Medium	32.6	24.2	31.9	
- High	61.0	70.6	63.4	
Living area (%)				0.145
- Rural	7.3	12.4	12.2	
- Suburban	58.9	53.6	58.0	
- Urban	33.7	34.0	29.8	
Meets PA guidelines (%)	33.4	63.4	48.9	<0.001*
Member of sport club (%)	63.9	78.4	67.2	0.006 *
How many friends do you have?	7.3 ± 10.5	9.2 ± 12.8	7.8 ± 10.5	0.211
Categories z-scores (BMI) (%)				0.047 *
- Underweight	4.2	4.1	7.9	
- Normal weight	86.4	91.2	58.7	

- Overweight	9.3	4.7	6.4	
PA behavior (min/week)				
Light PA (M±SD)	222.6 ± 197.6 [c]	261.5 ± 232.8	264.2 ± 223.0 [a]	0.019 *
Moderate-to-vigorous intensity PA (M±SD)	370.6 ± 348.1 [b,c]	585.8 ± 360.6 [a,c]	482.1 ± 385.3 [a,b]	<0.001*
Park use characteristics				
Park use (M±SD)	3.3 ± 1.7 [b,c]	3.8 ± 1.8 [a]	3.7 ±1.7 [a]	0.005*
Park duration (min/3 months) (M±SD)	71.7 ± 53.6 [b]	88.7 ± 68.8 [a]	82.9 ± 61.4	0.010*
Walking distance to closest park (min) (M±SD)	13.3 ± 11.7	13.6 ± 12.9	13.4 ± 12.1	0.973
Accompaniment to the park				
Friends (%)	62.4	73.3	60.2	0.022*
(Step)brother/sister/niece/nephew (%)	34.5	25.2	40.0	0.006*
Parents/grandparents/aunt/uncle (%)	33.8	17.0	38.4	<0.001*
Organised group (%)	21.6	14.8	16.6	0.134
Alone (%)	15.7	12.6	14.5	0.700
Dog (%)	12.9	10.4	14.5	0.464
Usual activities during park visitation				
Walking (%)	69.0	35.6	62.1	<0.001*
Ball sports (%)	31.4	71.9	42.4	<0.001*
Sitting/lying down (%)	43.6	24.4	36.5	0.001*
Jogging (%)	18.5	12.6	24.4	0.007 *
Active games (%)	10.5	5.9	20.1	<0.001*
Exercising (%)	5.9	10.4	15.9	<0.001*
Biking (%)	27.9	24.4	33.6	0.073
Standing (%)	15.0	12.6	18.0	0.268
Skating (%)	13.2	12.6	16.6	0.342
Yoga (%)	1.4	0.7	0.7	0.631
Usual transportation to parks				
Public transportation (%)	22.6	15.6	13.5	0.006 *
Walking (%)	72.8	70.4	69.0	0.541
Biking (%)	35.2	38.5	43.6	0.075
Car/motorbike as a passenger (%)	24.0	21.5	26.8	0.418
Skateboard/long board/scooter (%)	10.8	5.2	12.3	0.065

[a] significant difference with subgroup 1; [b] significant difference with subgroup 2; [c] significant difference with subgroup 3; * $p < 0.05$
